# Gray matter volume is associated with rate of subsequent skill learning after a long term training intervention

**DOI:** 10.1016/j.neuroimage.2014.03.056

**Published:** 2014-08-01

**Authors:** Cassandra Sampaio-Baptista, Jan Scholz, Mark Jenkinson, Adam G. Thomas, Nicola Filippini, Gabrielle Smit, Gwenaëlle Douaud, Heidi Johansen-Berg

**Affiliations:** a Oxford Centre for Functional MRI of the Brain (FMRIB), Nuffield Department of Clinical Neurosciences, University of Oxford, John Radcliffe Hospital, Headington, Oxford OX3 9DU, UK; b Mouse Imaging Centre, Hospital for Sick Children, 25 Orde Street, Toronto, Ontario M5T 3H7, Canada; c Department of Psychiatry, University of Oxford, Warneford Hospital, OX3 7JX UK; d NIMH, National Institutes of Health, Bethesda, MD 20892-1148, USA

**Keywords:** Structural plasticity, Skill learning, MRI

## Abstract

The ability to predict learning performance from brain imaging data has implications for selecting individuals for training or rehabilitation interventions. Here, we used structural MRI to test whether baseline variations in gray matter (GM) volume correlated with subsequent performance after a long-term training of a complex whole-body task. 44 naïve participants were scanned before undertaking daily juggling practice for 6 weeks, following either a high intensity or a low intensity training regime. To assess performance across the training period participants' practice sessions were filmed. Greater GM volume in medial occipito-parietal areas at baseline correlated with steeper learning slopes. We also tested whether practice time or performance outcomes modulated the degree of structural brain change detected between the baseline scan and additional scans performed immediately after training and following a further 4 weeks without training. Participants with better performance had higher increases in GM volume during the period following training (i.e., between scans 2 and 3) in dorsal parietal cortex and M1. When contrasting brain changes between the practice intensity groups, we did not find any straightforward effects of practice time though practice modulated the relationship between performance and GM volume change in dorsolateral prefrontal cortex. These results suggest that practice time and performance modulate the degree of structural brain change evoked by long-term training regimes.

## Highlights

• Inter-individual differences in brain structure correlate with subsequent performance outcome.
• Performance outcome plays an important role in positive structural brain change.
• Performance outcome and amount of practice modulate structural brain change.

## Introduction

As adults, we are often faced with the challenge of learning novel visuo-motor skills, such as using the touchscreen on a new smartphone. Intuitively, we might expect that how well we acquire these skills depend on multiple factors including how much practice we put in, and our individual aptitude for such learning. Studies in both animals and humans show that motor skill learning is associated with structural brain plasticity in the adult and during development (Draganski et al., 2004, Draganski et al., 2006, Hyde et al., 2009, Kleim et al., 1996, Scholz et al., 2009, Taubert et al., 2010). However, it remains unclear whether brain structural properties at baseline are associated with subsequent complex skill acquisition, and also whether the degree of brain structural change with long-term training depends on factors such as the amount of practice time and the performance outcome.

The ability to make predictions about an individual's future long term motor skill learning based on baseline brain structural characteristics could be applied in the context of talent identification (e.g. in elite athletes) and also has relevance in clinical scenarios, such as predicting response to the rehabilitation of movement abilities after brain damage. Inter-individual variability in human brain structure has been shown to correlate with variation in task performance in both expert and non-expert populations in cross-sectional studies (Gaser and Schlaug, 2003a, Gaser and Schlaug, 2003b, Johansen-Berg et al., 2007, Kuhn et al., 2012). However, studies that have tested whether baseline brain structural measures relate to subsequent behavioral response, have been limited to simple hand motor tasks and shorter time periods (between one and five training sessions) (Gryga et al., 2012, Tomassini et al., 2011). Here we test if baseline brain structure is associated with subsequent performance outcome with long-term training (several weeks) of a complex whole-body motor skill.

Evidence for a relationship between brain structural change and amount of practice or performance outcome is also limited. Amount of practice refers to the duration and or number of training sessions. Some studies have also examined practice density or intensity by defining a fixed number of training hours but not training sessions. Performance outcomes can be assessed by measuring performance after long-term training, average performance throughout training, or the rate of change in performance throughout the training period. While some previous studies have used fixed training schedules, others have allowed subjects to train at their own pace, or until they reach a particular performance criterion. In the context of juggling training, a popular experimental paradigm in this area, most studies have reported that brain structural changes are not correlated with how quickly subjects learn to juggle or how well they perform after training (Boyke et al., 2008, Draganski et al., 2004, Driemeyer et al., 2008, Scholz et al., 2009). One possibility is that structural changes reflect the amount of time spent training rather than training outcome; previous studies have predominantly used fixed amounts of training or fixed outcome criteria, making it difficult to tease apart effects of training time and performance outcomes. In the context of golf training, there is a recent evidence that higher training intensity, reflected in the number of days necessary to complete 40 h of training, results in greater gray matter increases, although no correlations with performance outcomes were reported (Bezzola et al., 2011).

The absence of a correlation between training outcome and structural changes in human neuroimaging studies is puzzling as functional plasticity and map reorganization as measured in animal studies seem to be associated with learning outcome rather than with amount of practice (Kleim et al., 1998, Plautz et al., 2000). It is not clear whether this apparent lack of a relationship between training outcome and structural brain change is real or reflects methodological factors. For example, the lack of an effect might be due to the assessment of the training outcome, i.e., the behavioral measures used might not be sensitive or might not represent the important aspects of learning that drive the structural changes.

In this study, we tested whether individuals' ability to learn a complex whole-body visuo-motor skill (juggling) could be explained by brain structural measures obtained before learning. We also tested if baseline brain structure was associated with brain structure change after long-term skill training. Furthermore, we varied the amount of training time in order to directly test whether an amount of practice or performance outcome modulates structural brain changes. To assess performance across the training period participants' practice sessions were filmed.

## Methods

### Participants

All subjects gave their informed consent to participate in the study in accordance with local ethics committee approval (REC B 07/Q1605/65).

44 participants with no prior experience of juggling were recruited and randomly assigned to one of the 2 groups: a high intensity training group that learned to juggle for 30 min per day, 5 days a week, for 6 weeks; and a lower intensity group that practiced for 15 min per day, 5 days a week, for 6 weeks. From the initial 44 recruited participants, 40 completed the study (22 in the higher intensity group and 18 on the lower intensity group) (mean age 23.8, standard deviation 3.5; 22 female). Participants were scanned at baseline, after 6 weeks of training and 4 weeks after the end of training. During the final 4-week interval participants were asked not to juggle.

All participants were right handed and matched for age and gender (low intensity group: mean age 23.8, standard deviation 3.3, 10 females) (high intensity group: mean age 23.9, standard deviation 3.6, 12 females).

### Behavioral assessment

**Fig. S1:**
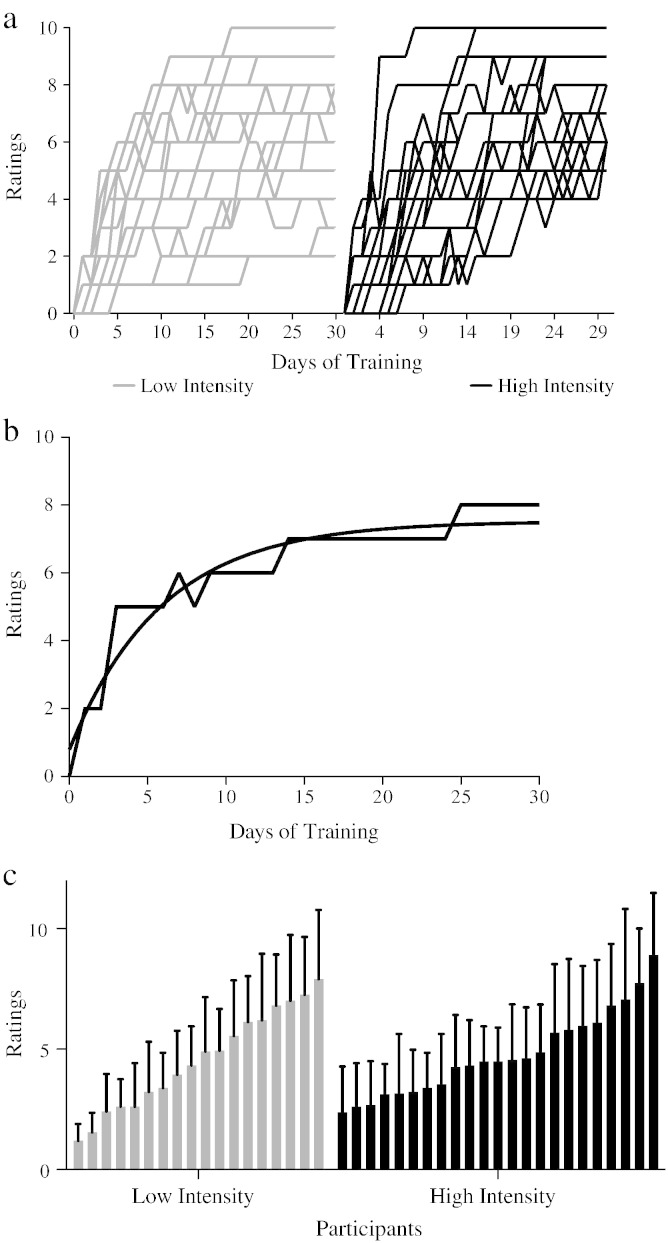
a) Learning curves for all participants. b) Learning and logarithm curve of a representative participant c) Average performance for all participants. Bars represent standard deviation.

Participants in the training groups had a group juggling lesson on the first training day, where the fundamentals of the 3-ball cascade were taught. Subsequently, participants were instructed to practice continuously for 15 (low intensity group) or 30 (high intensity group) minutes per day for 29 days. There was no fixed structure or number of juggling attempts per training session. Volunteers who mastered the ‘3-ball cascade’ before the end of the training period were encouraged to practice more advanced juggling patterns. After the training period, participants were not told to juggle for 4 weeks. Participants filmed every home training session using a webcam and were required to upload their training videos to a secure website daily. After the final scan (following the four-week period without juggling) participants were asked to film themselves again for 5 min while juggling. Videoing of training sessions ensured compliance and provided us with objective information for later assessment. For the daily performance scores the experimenter rated each of the 29 training videos per participant on a scale of 0–10 (Scholz et al., 2009) (0: 2 balls; 1: 1 cycle of ‘3-ball cascade’; 2: 2 cycles; 3: 3 cycles; 4: 5–10 s of sustained 3-ball cascade; 5: 10–20 s; 6: 20–30 s; 7:> 30 s; 8:> 60 s; 9:> 60 s and at least one other pattern for < 60 s; 10:> 60 s and at least one other pattern for > 60 s). A learning curve was plotted for each participant based on the score for each day. A logarithm curve was then fitted to each participant's learning curve and the slope of the curve (learning rate) was calculated (see Inline Supplementary Figure S1b).

Inline Supplementary Figure S1

For each subject we calculated the following measures: daily performance score, best performance score over all training days, performance on the last day of practice, average performance over 29 days, and learning rate. Furthermore long-term retention was calculated as the difference between the final behavioral test (4 weeks after participants stopped juggling) and the average performance of the last 3 days of juggling training. These scores were used to explore behavioral differences between groups.

We tested for performance differences over time and between groups with a Repeated Measures ANOVA (RM — ANOVA) of the daily scores including the factors of day (29 days of training) and group (high vs low intensity). When Mauchly's test of sphericity was statistically significant, Greenhouse–Geisser F-test was used and the respective degrees of freedom are reported.

Additionally, T-tests were used to investigate between-group differences in: performance on the last day of practice, best performance, learning rate, the last performance measure acquired after the last scan (4 weeks after participants were asked to stop juggling), and long-term retention. Of the several performance parameters calculated, average performance over 29 days per participant (from now on referred to as average performance or just performance) (mean = 4.83, SD = 1.78) was used to test for the effects of performance outcome on structural brain changes as this measure captured performance over the whole training period and showed a wide variation across subjects (See Inline Supplementary Figure S1c).

### MRI acquisition

Data was acquired on a 3 T Trio scanner (Siemens, Erlangen, Germany) with a 12-channel head coil. We acquired one axial T1-weighted anatomical image using a MPRAGE sequence (TR = 20.4 ms; TE = 4.7 ms; flip angle = 8°; voxel size = 1 × 1 × 1 mm^3^).

Two sets of whole brain diffusion weighted volumes (60 directions; b-value = 1000 s/mm^2^; 65 slices; voxel size 2 × 2 × 2 mm^3^; repetition time (TR) = 9.3 s; echo time (TE) = 94 ms) plus six volumes without diffusion weighting (b-value = 0 s/mm^2^) were also acquired. Due to technical problems DTI was only acquired in 35 participants (19 from the high intensity group and 16 from the low intensity group).

### MRI analysis

We carried out analyses with the FSL package, version 4.1 (http://fsl.fmrib.ox.ac.uk/fsl/fslwiki/). We analyzed T1-weighted anatomical images using FSL–VBM (Douaud et al., 2007, http://fsl.fmrib.ox.ac.uk/fsl/fslwiki/FSLVBM), an optimized VBM protocol (Good et al., 2001) carried out with FSL tools (Smith et al., 2004). A longitudinal protocol increasing the sensitivity for detecting changes in gray matter thickness was used to avoid any registration and interpolation bias (Douaud et al., 2009). In brief, the non-linear registration was determined from the gray matter images for all 3 time points averaged in midspace: this way, changes in gray matter detected between time points can be mainly interpreted as changes in thickness (Douaud et al., 2009, Scholz et al., 2009). More specifically, for each subject, we calculated the midspace between the 3 scans and aligned each scan to this midspace using linear registration (FLIRT) (7 DOF), after which all scans for a given subject were averaged in midspace. For each subject, we ran brain extraction with BET (Smith, 2002) on the midspace averaged brain and back-projected the brain mask to each of the original (native space) scans. Then the brain-extracted native space images were segmented into gray matter (GM) using FAST4 as the algorithm optimally works on non-interpolated data (Zhang et al., 2001). The resulting GM partial volume images were linearly transformed into that subject's midspace, averaged and then aligned to MNI152 standard space using the nonlinear registration FNIRT (Andersson et al., 2007a, Andersson et al., 2007b). The registered GM images (average across time for each subject) were then averaged across all subjects to create a GM study-specific template, to which each of the midspace average GM image was then non-linearly re-registered. Finally, we combined the affine midway transformation with the non-linear warpfield generated between the midspace averaged GM images and the study-specific template, and applied this final transformation to the original GM images for each scan. We modulated the registered maps using the Jacobian of the warpfield of the non-linear transformation from midspace to MNI study-specific template space to correct for local geometric expansions or contractions. The modulated segmented images were then smoothed with an isotropic Gaussian kernel with a sigma of 3 mm (~ 7 mm FWHM).

DTI data were analyzed with FMRIB's Diffusion Toolbox (FDT). First, all data were corrected for eddy current distortions and head movements. Voxel-wise maps of fractional anisotropy (FA) were then estimated for each subject and each time point using dtifit, and these were then analyzed using the Tract Based Spatial Statistics (TBSS) approach (Smith et al., 2006). Again to avoid any registration bias (Scholz et al., 2009), FA maps were registered for each subject to a midspace between scans and the registered maps were then averaged. The averaged maps for each subject were non-linearly aligned to FSL's standard FA template and averaged to generate a study specific mean FA map. The white matter skeleton was then extracted from this mean FA image by thresholding it at an FA value of 0.2 to represent the center of the tracts common to all subjects. The tract centers for each subject were projected onto the skeleton, which were then used for voxel-wise statistical comparisons.

### MRI statistical analysis

For statistical whole brain analyses of GM volumes and FA, a voxel-wise general linear model (GLM) was applied using permutation-based non-parametric testing (http://fsl.fmrib.ox.ac.uk/fsl/fslwiki/Randomize). Clusters were formed at t > 2 and tested for significance at p < 0.05, corrected for multiple comparisons across space (Nichols and Holmes, 2002). For GM analysis we are correcting across all GM voxels in the brain and for FA analysis across all voxels on the WM skeleton.

We tested whether baseline structural characteristics correlated with subsequent learning across all subjects. We addressed this question using GM or FA images from the baseline scan and analyzed this using a design matrix that included a regressor representing learning rate (slope), average performance and long-term retention as measurements of performance. Age and gender were included as covariates of no interest.

To address the effect of practice and performance on structural brain change with training, we have considered each pair of time points separately (scan 1 vs 2; 2 vs 3; 1 vs 3), creating difference images between time points for GM volume or FA, which were then analyzed separately with whole brain voxel-wise analyses. For each pair of time points, we used a single design matrix to model both performance and amount of training and the interaction between these variables. This allowed us to test for a main effect of time, as well as interactions between performance, practice, and both in a single design.

## Results

### Behavioral results

**Fig. 1:**
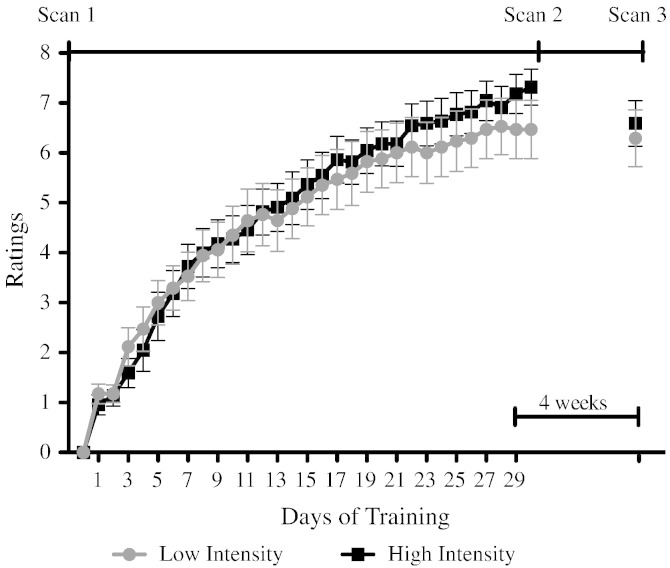
Average performance score for each group per day. (0: 2 balls; 1: 1 cycle of 3-ball cascade; 2: 2 cycles; 3: 3 cycles; 4: 5–10 s of sustained 3-ball cascade; 5: 10–20 s; 6: 20–30 s; 7: > 30 s; 8: > 60 s; 9: > 60 s and at least one other pattern for < 60 s; 10: > 60 s and at least one other pattern for > 60 s). There is a significant effect of day but no significant interaction effect or significant differences between groups. Bars represent standard error.

Participants were asked to practice juggling, while filming themselves, 5 days a week for 6 weeks, resulting in a total of 30 days training and 29 videos (excludes the juggling class on the first day of training). Compliance was good: Subjects submitted on average 27.9 videos (SD = 2.2) and reported juggling on average for 28.6 days (SD = 0.9). The low intensity (15 min daily practice) group showed a mean improvement in performance (i.e., from a starting point of zero to their final day performance scores) of 6.47 rating points (SD = 2.4), and the high intensity (30 min daily practice) group showed a mean improvement in performance of 7.3 rating points (SD = 1.7) but the difference in performance improvement between groups was not statistically significant (t_(38)_ = 1.29, p = 0.205). We explored differences between groups and over practice days with RM — ANOVA including factors of day (29 days) and group (high vs low intensity) (Fig. 1a). The test revealed an effect of day on performance (F _(4.127, 152.704)_ = 157.5, p < 0.001), confirming significant improvements in performance with practice. However, no interaction effect between day and group (F _(4.127, 152.704)_ = 1.113, p = 0.353) or main effect of group (F _(1, 38)_ = 0.064, p = 0.801) was found. There were no differences found between groups for the average performance over 29 days (t_(1, 38)_ = 0.077, p = 0.783), learning rate (slope) (t_(1, 38)_ = 1.47, p = 0.234) or best performance (t_(1, 38)_ = 1.439, p = 0.238). Differences between groups were also tested in the last performance measure acquired after the last scan (4 weeks after participants were asked to stop juggling). There were no significant differences between groups at this last time point (t_(1, 38)_ = 0.411, p = 0.683). We did not find significant differences between groups for the long-term retention (t_(1, 38)_ = 1.52, p = 0.141). In summary, daily practice improved juggling performance but the amount of practice per day did not have any statistically significant effect on performance outcomes.

### GM volume at baseline correlates with how fast participants learned to juggle

**Fig. 2:**
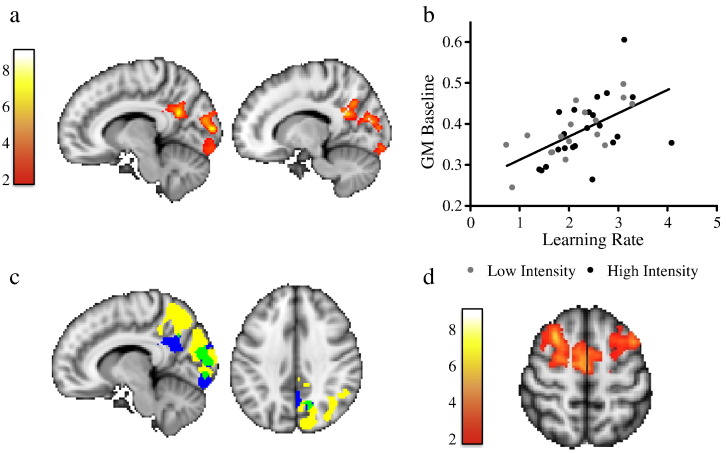
Baseline GM volume correlates with subsequent learning rate. a) GM volume in right visual and parietal cortex at baseline correlates with subsequent learning rate. Yellow–red voxels represent significant clusters superimposed on MNI template. Color bar represents t-scores. b) Scatter plot showing the correlation between GM volume averaged across voxels in significant brain areas (shown in 2a) and learning rate for the low intensity group (dark gray symbols) and the high intensity group (black symbols) is displayed for visualization of the range of individual values only and not for inference. c) Regions where GM volume correlates with subsequent learning partly overlap with regions where GM changes with learning in the current study (see Fig. 4c). Yellow cluster corresponds to regions showing significant GM volume change after learning (from Fig. 4c), blue cluster represents regions showing a correlation between GM volume at baseline and learning rate (from Fig. 2a) and green cluster shows the intersection between both clusters. d) GM volume in bilateral DLPFC and SMA correlated with long-term-retention. Yellow–red voxels represent significant clusters superimposed on MNI template. Color bar represents t-scores. Clusters are shown at a corrected cluster extent threshold of p < 0.05.

We found that GM volume at baseline correlated with subsequent learning rate. Participants with higher GM volume in right visual areas (including areas V1, V2, V4, according to the Juelich probabilistic atlas http://fsl.fmrib.ox.ac.uk/fsl/fslwiki/Atlases), right precuneus and right posterior cingulate at baseline learned to juggle faster than jugglers that had lower GM volume in these regions (cluster p = 0.05) (Fig. 2a). Interestingly, some of these brain areas partially overlap with regions that show GM change with learning in the same participants (described below Fig. 4c) and in a previous related study (Scholz et al., 2009), particularly in precuneus, V1 and V2 areas (Fig. 2c). We tested whether GM volume at baseline was associated with the magnitude of GM change in these overlapping regions, but no correlation was found.

Furthermore, we found a positive correlation with GM volume in the DLPFC and SMA and long-term retention (cluster p = 0.02) (Fig. 2d). We did not find any negative correlations between GM and performance measures.

We found no evidence that baseline FA was related to later performance at the selected threshold (p < 0.05). There were no significant differences between groups in baseline brain structure.

### What drives structural brain changes? Effects of time, amount of practice or performance?

In order to distinguish the contribution of time, practice and performance outcome to structural brain plasticity a series of whole brain analyses were performed, in which the inputs were GM or FA difference images between each pair of time points (1 vs 2; 1 vs 3; 2 vs 3), and the regressors represented factors of practice (low intensity vs high intensity groups) and performance outcome (average performance over 6 weeks of training). For each pair of time points, the main effect of time was tested. Additionally, interactions were also tested between time and performance; time and practice; and time, practice and performance.

### Training period: changes between scan 1 and scan 2

**Fig. 3:**
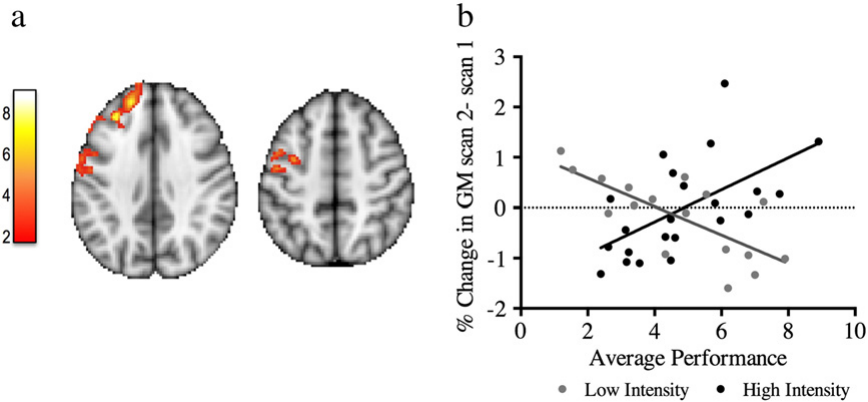
Interaction effect between practice group and average performance, between scan 1 and scan 2. a) Yellow–red voxels correspond to the significant cluster, superimposed on MNI template. Color bar represents t-scores. b) Scatter plot of mean GM change and average performance correlation for the low intensity group (dark gray symbols) and the high intensity group (black symbols) are displayed for visualization of the range of values only and not for inference. Clusters are shown at a corrected cluster extent threshold of p < 0.05.

No main effect of time was found, and no two-way interactions between time and performance or time and practice on GM volume differences between scans 1 and 2. However, a significant interaction between time, performance and practice was found in the left motor and dorsolateral prefrontal cortex (DLPFC) (p = 0.05), demonstrating that the relationship between GM changes over time and performance varied between the two practice groups (Fig. 3). The different relationships underlying this interaction can be seen in the scatter plot in Fig. 3: For the low intensity group, better average performance was negatively associated with changes in GM volume between scans 1 and 2 in left motor and DLPFC, while for the high intensity group, better average performance was positively associated with changes in GM volume between scans 1 and 2 in the same areas.

No main effect of time, and no interactions between time and practice group were found for scans 1 to 2 for FA. A trend for an interaction between time and performance was found for FA change in the posterior region of the corpus callosum (p = 0.08), reflecting greater increases in FA for subjects showing greater increases in FA between scans 1 and 2 in this region.

### Longer term effects: scans 1 to 3; scans 2 to 3

**Fig. 4:**
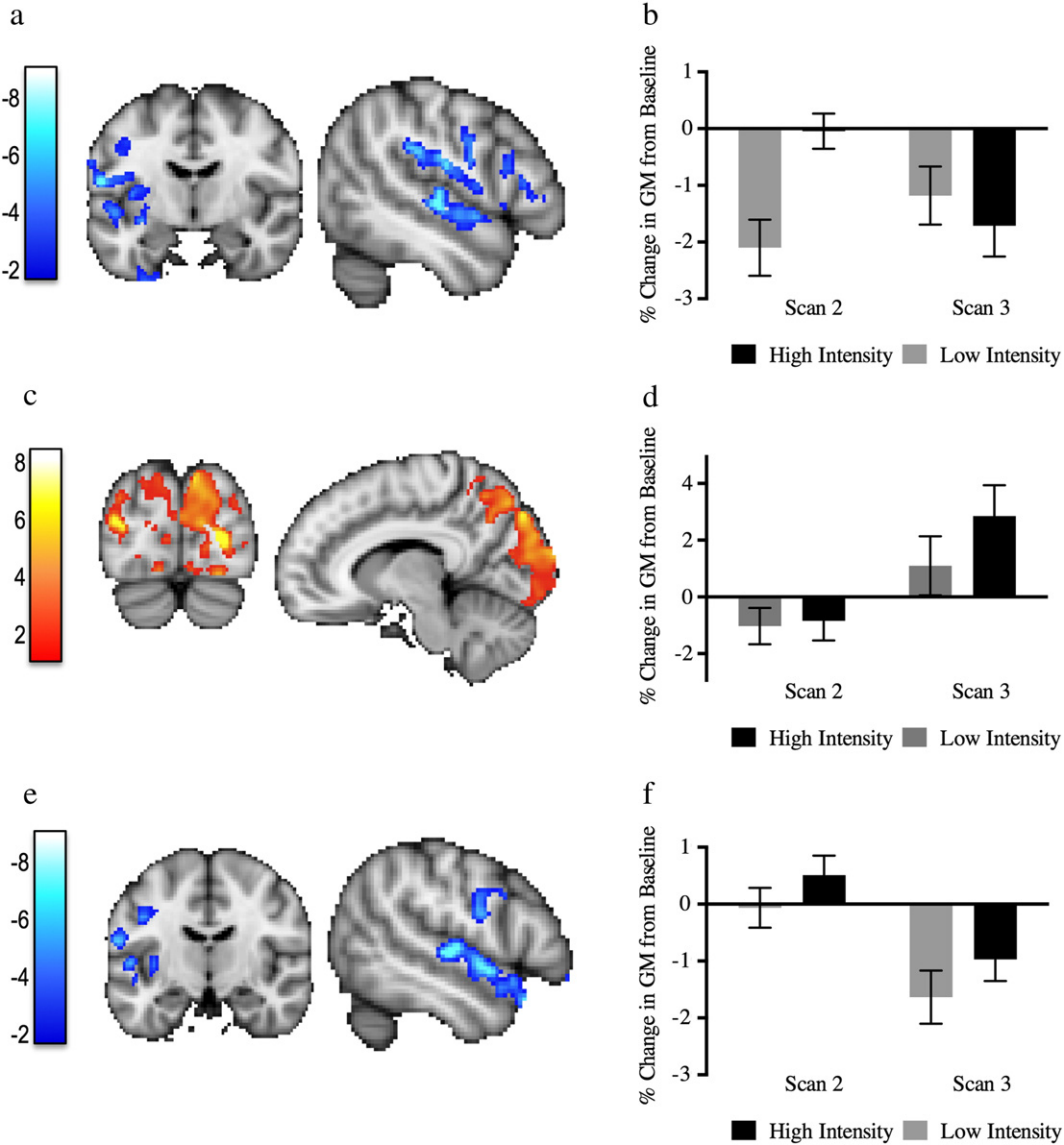
Longer term effects: Scans 1 to 3 and scans 2 to 3. a) GM volume decreases between scans 1 and 3 in the left temporal cortex, insula and operculum. Blue–dark blue voxels correspond to the significant clusters. b) Mean GM values of the blue clusters throughout time relative to scan 1. c) GM volume increases between scans 2 and 3 in the visual and parietal cortex (Yellow–red voxels). d) Mean GM values of the yellow–red clusters at different time points relative to scan 1. e) GM volume decreases between scans 2 and 3 in the superior temporal gyrus, insula and operculum (Blue–dark blue voxels). f) Mean GM values of the blue clusters throughout time relative to scan 1. Plots are for illustrative purposes only and not for inference. Error bars represent standard error. Clusters are superimposed on MNI template. Color bars represent t-scores. Clusters are shown at a corrected cluster extent threshold of p < 0.05.

A main effect of time on GM volume changes between scans 1 and 3 was found that corresponded to decreases in GM in the left operculum, insula and superior temporal gyrus (p < 0.028) (Figs. 4a, b). We did not find any interactions with performance for this pair of time-points.

During the follow-up period (between scans 2 and 3) we found two main effects of time. Jugglers had increased GM volume in occipital and parietal regions bilaterally (p = 0.015) (Figs. 4c, d), in similar areas to those reported by the previous studies to change with juggling training (Boyke et al., 2008, Draganski et al., 2004, Driemeyer et al., 2008, Scholz et al., 2009). Decreases in left operculum, insula and superior temporal gyrus were also found (p = 0.05) (Figs. 4e, f).

There were no significant main effects of time, or interactions of time with performance, or practice for FA. There were, however, trends for a main effect of time between scan 2 and scan 3 (p = 0.09) and scan 1 and scan 3 (p = 0.07) in the corticospinal tract, motor and premotor areas of the corona radiata, reflecting increases in FA over time in these regions.

**Fig. 5:**
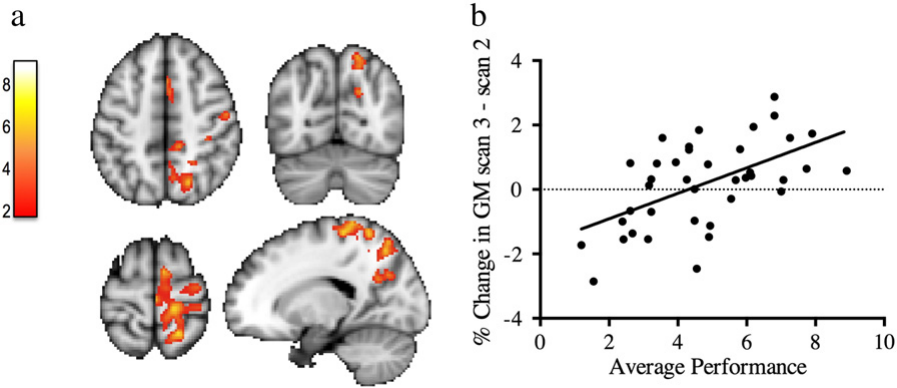
Participants with better performance have higher GM increases during the follow-up period. a) Cluster's mean GM change values correlation with the average performance after the learning period (between scans 2 and 3). b) Correlation plot between the average performance and GM change in a) is displayed for visualization of the range of individual values and not for inference. Color bar represents t-scores. Clusters are shown at a corrected cluster extent threshold of p < 0.05.

There was also an interaction between time and performance for GM change for this time period (between scans 2 and 3): jugglers that had higher GM increases in the right motor cortex, parietal cortex and pre-SMA also scored a higher average performance during their learning period (p = 0.015) (Fig. 5) There were no interactions with practice group.

## Discussion

We found that inter-individual differences in GM volume in occipital-parietal areas before learning correlated with subsequent learning rate in a complex visuo-motor task. The identified brain areas have been previously implicated in the present task as part of a large GM cluster that was found to increase in volume after juggling learning (Scholz et al., 2009). The same areas overlapped with a significant increase in GM volume after learning in the present group of participants. Additionally we directly tested for performance and practice effects on structural brain change. We found that performance outcome plays an important role in modulating positive structural brain change over certain time points in GM volume. We did not find straightforward effects of amount of practice on structural brain change, but found evidence to suggest that an amount of practice interacts with performance outcome in modulating structural brain change, as discussed below.

### GM volume at baseline correlated with how fast participants learned to juggle

GM volume at baseline in occipital-parietal areas before learning correlated with subsequent learning rate. These areas have been previously implicated in visual spatial processing and spatial attention processes that would be important for learning to juggle. More specifically, the posterior cingulate cortex (PCC) is involved in spatial attention shifting and in navigation tasks (Hopfinger et al., 2000, Kovacs et al., 2010). The precuneus is a major association area that connects to other parietal regions such as the intraparietal sulcus (IPS) as well as premotor regions like the supplementary motor areas (SMA) (Cavanna and Trimble, 2006). Due to its anatomical connections, this area has been considered to be part of a network that specializes in spatially guided behavior and in the spatial relationships for body movements, playing a role in the visual guidance of hand movements, reaching and grasping (for review see (Culham and Valyear, 2006)). This region is also more active in complex bimanual tasks compared to a complex unilateral task (Wenderoth et al., 2005). Taken together, all these areas are functionally relevant for juggling, which involves complex bilateral reaching and grasping movements. It is feasible that people who have greater eye-hand coordination, that translates into higher GM volume (either by previous learning or genetic predisposition) in brain areas that are related to this function, learn to juggle faster.

Furthermore, we found a positive correlation with GM volume in bilateral DLPFC and SMA and long-term retention measures. Participants that have greater GM volume in these areas involved in complex whole-body movements and bilateral movements were found in this study to retain the acquired skill for longer.

Most studies that have examined the relationship between behavior and brain structure in non-expert populations have used simple tasks that can be performed with more or less difficulty by the participants (Johansen-Berg et al., 2007, Rudebeck et al., 2009, van Gaal et al., 2011). However, in this study the participants were scanned before they were able to juggle. The observed correlation between baseline brain structure and subsequent learning rate in this complex task shows that the potential to learn complex skills partially depends on structural properties of gray matter of functionally relevant areas. Similarly, a previous study of sequential pinch force learning found that baseline GM volume in the cerebellum predicted subsequent behavioral gains (Gryga et al., 2012). That same study found that baseline cerebellar structure also predicted structural change in motor, premotor and prefrontal cortex. By contrast, in the current study we found that even though there was overlap between regions whose baseline structure predicted learning and regions showing increase in GM volume after learning, the magnitude of subsequent GM change after learning was not associated with GM volume at baseline.

The observed correlations between baseline GM structure and subsequent learning should motivate future studies to test the predictive value of baseline GM structure. The ability to predict subsequent learning from baseline imaging measures is relevant to a number of real-life situations. For example, it can offer the opportunity to channel time-consuming training and limited resources by identifying people that might benefit the most from it. Such an approach could be used in the context of elite sports and highly skilled professions, as well as in clinical domains such as predicting response to rehabilitation. This seems to be true not only for motor skills but also for the cognitive domain. Recently a study found that baseline hippocampal volume in third grade students was correlated with behavioral improvements after one-on-one math tutoring (Supekar et al., 2013). This knowledge could be used to tailor training programs to people's needs, not only taking advantage of the “natural” inclinations but also maximizing brain plasticity mechanisms through learning.

### Effects of time, amount of practice or performance on gray matter change

In some regions and time periods, GM volume was not associated with performance and/or amount of training. Specifically, we found bilateral GM increases in widespread regions of visual and parietal cortex, during the follow-up period, in regions that coincide with the previous reports of GM change with juggling (Draganski et al., 2004, Scholz et al., 2009). However, the increases detected in the current study were found at a later time-point compared to the previous studies.

We were particularly interested in how changes over time varied with practice time and performance outcomes. We found that participants with better performance had higher increases in GM volume during the period after training (i.e., between scans 2 and 3) in dorsal parietal cortex and primary motor cortex. These regions are relevant to the trained skill as they are involved in complex motor learning and eye-hand coordination (Culham and Valyear, 2006). This finding apparently contradicts some previous studies where no correlations between GM structural change and performance were found (Draganski et al., 2004, Driemeyer et al., 2008, Scholz et al., 2009). There are however a number of differences between the current study and previous reports that could help explain this discrepancy. For example, in the current study, the correlation related to structural brain changes detected during the follow-up period between scans 2 and 3, and might therefore be related to delayed, off-line processes rather than changes occurring during the training period itself, which was the focus of most prior studies. Furthermore, for the first time video recording was used in the current study to objectively assess daily performance, which could have provided a more sensitive or accurate measure than the self-report used in a similar previous study (Scholz et al., 2009). Finally, whereas many prior studies (Boyke et al., 2008, Draganski et al., 2004, Driemeyer et al., 2008) required participants to reach a set performance criterion during the training period, we did not impose any criteria, and we also deliberately varied practice time, and so ended up with a larger spread of performance across individuals.

Our findings of correlations between brain change and behavioral change are consistent with a previous study of sequential pinch force learning which found that subjects showing greater performance improvements showed training-related increases in GM in motor, premotor and DLPFC, whereas those with smaller behavioral gains showed no change or a decrease in GM with training in the same regions (Gryga et al., 2012). In the current study, we also detected variations with performance in DLPFC, a region that is involved in effortful motor learning (Jueptner et al., 1997, Sakai et al., 1998). In addition, by manipulating practice time in our experimental design, we were able to show that our observed relationship between performance and training-related brain changes in DLPFC also varied between the high and low intensity practice groups: participants that perform better either increase (for the high intensity group) or decrease (low intensity group) their GM after learning.

While the specific relationships between practice, performance and brain change found here are complex, we believe that some aspects of our results can be better understood when considered in the light of animal literature concerning potential underlying cellular mechanisms, as discussed below.

### Underlying cellular mechanisms

Because MRI does not allow us to differentiate the underlying cellular mechanisms, it is not straightforward to interpret the structural changes detected here. For example, increases or decreases in GM volume can be attributed to a variety of events, such as myelination, angiogenesis, synaptogenesis, spine formation or elimination, dendritic branching or pruning, gliogenesis and even neurogenesis (Zatorre et al., 2012). There is extensive evidence in animal literature that all these events occur in response to experience and learning (Anderson et al., 1994, Chang and Greenough, 1982, Eriksson et al., 1998, Gould et al., 1999, Greenough et al., 1985, Hihara et al., 2006, Ramirez-Amaya et al., 2001).

Evidence from animal studies also suggests possible interpretations for some of the more counter-intuitive findings from the current study. For example, similar to previous human imaging studies (Gryga et al., 2012, Langer et al., 2012, Taubert et al., 2010), we found both decreases and increases in brain structural measures in task-relevant areas, some of which were modulated by practice or performance. Evidence from several animal and human studies suggests that the time-course of GM structural brain change is not linear (Kleim et al., 2004, Taubert et al., 2010). Therefore, different phases of learning might be associated with different cellular mechanisms that MRI is not able to distinguish. For instance, an initial learning phase might be associated with rapid and transient GM remodeling in functionally relevant brain areas, whereas more persistent GM changes might be associated with later learning phases, consolidation and long-term storage. Animal studies have provided evidence that this is indeed the case. Synaptogenesis and map reorganization occur during the late phase in a rat reaching task, but not during the early learning phase, with synaptogenesis preceding map reorganization (Kleim et al., 2004). Recent studies employing invasive in-vivo techniques have shown that new spines formed within hours, that were relevant for task acquisition, are stable, while learning destabilizes older spines that are later eliminated (Xu et al., 2009, Yang et al., 2009). Spine formation and elimination were found to correlate with performance (Yang et al., 2009). These studies provide evidence that not only spine formation but also spine elimination is important for coding learning and performance, thus circuit pruning is an essential aspect of neural plasticity. Due to the technique employed, these studies are not able to track changes in the whole dendritic tree, however there is evidence from histological studies that dendritic trees can be remodeled by experience (for review see (Markham and Greenough, 2004)).

Although spine formation and elimination of old spines occur during early learning, with a small number of new spines persisting throughout life (Yang et al., 2009), it is not clear what happens at the structural level during later stages of learning. There is evidence that map reorganization, such as enlargement of the hand representation with motor skill learning (Kleim et al., 1998), occurs in late stages preceded by synaptogenesis (Kleim et al., 2004), but whether the map reorganization is sustained by synaptogenesis and functional changes alone or by more complex structural reorganization is unknown. Also unclear is the time-course of non-neuronal events, like angiogenesis or gliogenesis, which might more realistically underlie some of the MRI detected GM changes. Furthermore, late stages in most animal studies usually correspond to a few days or weeks. Learning stages will be intrinsically connected to the type and complexity of the task; a week can be enough to master a simple finger tapping sequence but it will take months or years to learn to play a complex piece of music on the piano. It is likely that the more complex the skill, and the longer it takes to master, the greater the brain restructuring needs to be.

These animal studies highlight the fact that learning encompasses circuit refinement, thus including pruning as well as formation of new connections. This could explain why decreases in GM volume were found in task relevant brain areas. Furthermore, the observed interaction between practice group, performance and time in the DLPFC could reflect the different practice groups being at different stages of learning, despite the apparent absence of performance differences. It is conceivable that cellular mechanisms are dependent on the practice level — from our findings we would anticipate that lower amounts of practice would elicit pruning and rely mostly on previously established functional connections, whereas higher amounts of practice would cause formation of new connections.

### Limitations

We did not find performance differences between naïve participants that learned to juggle for 15 min per day and participants that practiced twice as much, for the same period of time, but it is possible that our measures are too crude to detect subtle differences between groups. We only assessed the amount of sustained juggling each participant could perform in each training session and did not quantify juggling speed or more importantly the quality of the movement. Similarly, assessing changes in performance within each training session could provide more sensitive measures of group differences. Muscle fatigue, concentration or memory interferences are other temporary factors that might have affected the high intensity group immediate performance thus obscuring any differences in performance between groups (Kantak and Winstein, 2012). Despite this, we were still able to find a relationship between a derived performance outcome and brain structure change.

Furthermore, we did not assess the previous experience in complex visuomotor skills such as sports and musical training. However, we did not find any differences in brain structure between groups at baseline and the participants were comparable in terms of baseline demographics.

We have reported correlations between baseline GM measures and subsequent learning behavior. However, to directly test the ability of baseline GM characteristics to predict learning behavior on an individual subject basis would require a stricter test, such as out-of-sample cross validation. Unfortunately, our current sample was not large enough, considering the variance in the data, to robustly obtain high accuracy with such cross-validation approach.

For our longitudinal analyses, we calculated difference maps between each pair of scan time points (i.e., 1v2, 2v3, 1v3) and fed these into separate general linear models. However, we did not correct the subsequent contrasts for these three separate measures. The main effect of time corresponding to GM increases (Fig. 4b), and the correlation between GM change and performance (Fig. 5) would have survived such correction, whereas the interaction with practice group (Fig. 3) and the GM decreases (Fig. 4c), would not. Therefore the reported interaction with practice group and the decreases should be considered exploratory findings.

We did not detect statistically significant effects of time, practice or performance on FA, though a few trends for increasing FA with training and with greater behavioral outcomes were found in task-relevant pathways. The lack of significant effects is likely due to the smaller number of participants acquired with DTI protocol compared with the T1-weighted scan from the current study and compared with the previous studies that have reported FA change with visuomotor training (Scholz et al., 2009, Taubert et al., 2010).

Recently, concerns have been raised about the current evidence from MRI studies supporting structural plasticity in the adult human brain (Thomas and Baker, 2013). The authors mainly discuss flaws in the experimental design, statistical and analysis methods, reproducibility of the findings, and the relationship between the findings and behavioral performance (Thomas and Baker, 2013). The current study did not set out to demonstrate structural change per se, but rather to identify factors that might modulate the degree of structural change (i.e., practice time and performance outcome). In this study we have addressed some of the methodological concerns raised by using unbiased mid-space registration (as developed in (Douaud et al., 2009) and used in a previous juggling study by (Scholz et al., 2009)), robust statistical analysis and have found a correlation between GM volume increases and behavioral performance.

## Conclusions

Individual differences in brain structure in areas that are functionally relevant to motor skill learning relate to the speed at which a novel visuo-motor skill can be acquired. Furthermore, learning the skill itself drives structural changes that depend to some extent on training outcomes and practice time. The current study suggests that MR-detectable changes in brain structure with learning are more complex than previously thought. Although we can only speculate about the underlying cellular mechanisms, the specific relationships reported here are consistent with the animal studies of dynamic, bi-directional structural changes with learning.

## Funding

This work was supported by the 10.13039/100004440Wellcome Trust (WT090955AIA to H J-B) and 10.13039/100006129FCT (SFRH/BD/43862/2008 to C S-B). GD is supported by an 10.13039/501100001322MRC Career Development Fellowship (MR/K006673/1). The research was further supported by Marie Curie Actions (Adaptive Brain Computations network PITN-GA-2008-290011) and by the 10.13039/501100000272National Institute for Health Research (NIHR) Oxford Biomedical Research Centre based at Oxford University Hospitals NHS Trust and University of Oxford. The views expressed are those of the author(s) and not necessarily those of the NHS, the NIHR or the Department of Health.

## Conflict of interest statement

No conflicts of interest are reported.
